# Can we trust measures of trust? Measurement invariance in trust in EU news media

**DOI:** 10.1007/s43545-022-00534-1

**Published:** 2022-10-07

**Authors:** Eliyahu V. Sapir

**Affiliations:** Department of Political Science, Maastricht University, Maastricht, The Netherlands

**Keywords:** Trust in news media, Measurement invariance, Multi-group confirmatory factor analysis, Cross-context comparison, EU

## Abstract

Trust in the news media received wide scholarly attention for almost a century, which was further boosted as a result of recent developments in the media landscape and changes in how news is made and consumed. Despite that, the conceptualization of trust in the news media is still debated, and its measurement comparability has not yet been established. In this paper, I build up on earlier conceptualizations of trust in the news media, and test three theoretically derived measurement models to determine their cross-cultural equivalence in 28 EU countries. Using Eurobarometer data, I test the validity and comparability of these measurements employing multi-group confirmatory factor analysis. The findings indicate that trust is a unidimensional latent construct, equally interpreted across contexts. People's level of trust in the news media reflects their general attitude to the news stories and reporters in all sources of media they are exposed to. While bifactorial measurements of news media trust, differentiating between legacy and online sources, have some merit in single case-studies, they are non-invariant and therefore non comparable. This means that any cross-population differences found employing them are likely a function of measurement idiosyncrasies or other unknown factors.

**Supplementary Information:**

The online version contains supplementary material available at 10.1007/s43545-022-00534-1.

## Introduction

It has long been recognized that the extent to which citizens trust political and social institutions is of vital importance to the stability and performance of democratic political systems and modern societies (Almond and Verba 1963; Hooghe 2011). Some scholars see trust in institutions as a prerequisite for the very survival of democratic regimes (Norris 1999), while others perceive it as a means for preventing seismic changes in the workings of representative democracies (Klingemann and Fuchs 1995). There is an agreement in the literature that trust in political institutions indicates that members of the political community ‘feel that their own interests would be attended to, even if the authorities were exposed to little supervision or scrutiny’ (Easton 1975, p. 447).

Trust in social institutions has been similarly acknowledged as essential for the functioning of a diverse, modern society, as a means for promoting social cohesion, integration and stability (Kohring and Matthes 2007). The importance of one institution, namely the news media, was recognized early on, as central to the functioning of democracy. It provides the means by which citizens become informed and learn about their community, its problems and the various, often competing ideas for addressing these problems. Having this information at their disposal, citizens can lead their lives in a free and self-governing fashion (Strömbäck et al. 2020). Furthermore, they need to have confidence that other members of their community are similarly informed. Unless they can trust the news media to deliver the required common knowledge, they are less likely to trust reasoned political decision-making, or to accept political decisions (Coleman 2012; Tsfati and Cohen 2005).

With an emerging sense of a crisis in public trust in the news media and other social institutions (Bogaerts and Carpentier 2013; Coleman 2012) and reports on their historically low levels (Fisher 2018), researchers must have a valid way of gauging people’s trust in this specific social institution, to allow their cross-time and cross-case comparisons to be meaningful. Short of such a valid measure, trust in the news media may be a construct conditioned by time/case context and insufficiently independent from other constructs. This issue is particularly relevant in today’s news media landscape, with the considerable shifts in how news is made and consumed through a myriad of legacy and alternative sources (Kiousis 2001). Establishing the cross-context equivalence of such a measure is a prerequisite for any comparative analysis employing it. Surprisingly, and despite nearly a century-long interest in trust in news media, there is no common definition of what precisely this concept entails (Fisher 2018), nor is there an agreement on its operationalization, the specific media sources it should pertain to, or their relative weight and overall metric dimensionality.

This means that determining the meaning of observed cross-case differences is still impossible, as comparing the levels of news media trust across individual cases can only be possible if they are measured validly and invariantly (Medina et al. 2009). If such equivalence is not established, any cross-context differences may reflect measurement idiosyncrasies or other unknown factors rather than actual variances, and would therefore be biased. The purpose of this paper is to develop a valid way to measure the level of trust that citizens of 28 EU member states have in the news media, by establishing its cross-context invariance, which would provide the required evidence to indicate that it captures identical aspects of the latent trust construct. Since previous, comparative research relies primarily on survey measures of trust in the news media, the specific motivation in this paper is to understand to what extent can standard ‘trust in media’ survey indicators represent a single construct of trust in the news media, that will be transferable across diverse societies.

The paper proceeds as follows. First, a review of the literature on trust in the news media will be presented, to identify various conceptualizations, operationalizations and analytical perspectives employed to measure this construct. Measurement invariance will be introduced next, as a method that enables determining the validity of different measures of the latent construct. The data and case selection employed in the analyses will be discussed next, followed by specifying three hypothesized measurement models. The analysis will be completed by testing the invariance of these competing measurements of trust in news media and will be followed by a discussion of the findings with concluding remarks and recommendations for future research.

## Trust in news media

The concept of trust has received wide research attention across various disciplines, including political science, sociology, anthropology, psychology, philosophy, economics, marketing, management studies and more (Bauer 2021). Trust is one of the enabling forces identified as necessary for all social activity and interactions (Delhey and Newton 2003). At its core, trust denotes a relationship between a *trustor*, who is willing to engage in action based on positive expectations about the *trustee*, the recipient of trust (Barber 1983). Trust situations are inherently asymmetrical, as the trustee holds the resources or competences, and the trustor cannot acquire them without cost. This asymmetry leads to uncertainty and risk to the trustor, who must voluntarily relinquish control, sometimes beyond the level warranted by circumstances (Yamagishi and Yamagishi 1994). In sum, trust is the willingness of the trustor ‘to be vulnerable to the actions of another party based on the expectation that the other will perform a particular action important to the trustor, irrespective of the ability to monitor or control that other party’ (Mayer et al. 1995, p. 712).

An additional layer of complexity is introduced when considering the news media as the object of people’s trust. This is because individuals may have varying levels of trust in news messages, sources and journalists, and news organizations or groups thereof, as well as a general perspective on the media as a whole. The asymmetry and risk are clear. News media users do not possess the competences to meticulously verify the credibility of news content and often try to find some clues to legitimate their trust and to mitigate the inevitable risk (Strömbäck et al. 2020; Tsfati and Cohen 2005). They do so, based on believing that the media will perform in line with their expectations (Hanitzsch et al. 2018).

People’s trust in the media received scholarly attention for the better part of the twentieth century (Kiousis 2001). The interest only increased at the turn of the century, with global technological, economic and social changes, and even further with the move to Web 2.0, which introduced user-generated information and an unprecedented competition for people's attention from a myriad of alternative news sources (Newman et al. 2018; Strömbäck et al. 2020). Research on media trust regularly uses the terms trust and credibility interchangeably (Kiousis 2001; Kohring and Matthes 2007). This research can be grouped into three groups, based on the leading perspectives on the media’s performance at the micro, mezzo and macro levels.

Earlier research took a micro-level perspective and focused on people’s trust in the source of information. Pioneering research into source credibility conducted at Yale was based on earlier research on prestige (Arnett et al. 1931). It tried to establish that on top of the content of any message, the source that delivers it determines how it will be received by the public. The main finding was that perceptions of expertise and trustworthiness of the source, whether a person or an organization, influenced people's willingness to trust the message and change their minds on various issues (Hovland et al. 1953). Focusing on the source was criticized for lacking a robust theoretical base, as the Yale team failed to specify the core dimensions that cause people’s trust (Kiousis 2001). It was further unclear whether attributes identified in their research such as ‘expertness’ were trust dimensions or merely its correlates (Kohring and Matthes 2007).

Another micro-level line in research has explored the credibility of messages regardless of their source. This focus is preferred by many communication-studies scholars who view people's trust in the news media as their affirmation that news media reporting is done professionally, fully, accurately and fairly (Strömbäck et al. 2020; Tsfati and Cappella 2003). Various attributes have been identified as the basis for message trust, including safety, qualification and objectivity (Kiousis 2001), completeness, conciseness, consistency, representativeness, accuracy, authenticity and believability (Appelman and Sundar 2016). Other analyses have found that credibility of messages is associated with their structure and content (McCroskey and Mehrley 1969; Metzger et al. 2003).

The problem with this perspective is that it is similarly unclear whether these attributes are independent and comparable across different contexts. It is also unclear to what extent can the message be separated from the source that delivers it and the medium it is delivered through (Metzger et al. 2003). In addition, is it reasonable to expect the public to be able to determine the quality of news media reporting and to decide whether or not it is trustworthy (Strömbäck et al. 2020)? If people do possess these abilities, then why is the media needed at all? And what precisely is the risk these informed citizens take in trusting the news media? Lastly, if people constantly engage in a calculus to assess the quality of every piece of information reported by the media, we should expect a high volatility in any measure of media trust, especially considering the rich modern media landscape. Yet media trust is stable across time (Ladd and Podkul 2019), which indicates that it is not limited to specific sources or messages.

At the mezzo-level, researchers have tried to understand media credibility by understanding public trust in a particular news medium, such as newspapers, radio or television. Already in the 1930s, when the print industry became concerned with the effects of the popularization of news radio on readership and advertising revenues, it started studying and comparing different media credibility (Self and Roberts 2019). In the 1950s, it was the radio industry that became wary of the spread of television and conducted similar studies to assess and compare the credibility of different media (Newhagen and Nass 1989). In the 1970s, the US Television Information Office commissioned surveys about the credibility of different media, assuming their messages were inconsistent (Roper 1969). In recent years, many surveys collected data on attitudes toward online news, social media and video hosting platforms to complement the battery of media items and understand people’s attitudes toward them (Newman et al. 2018).

While this information is useful, it has three main shortcomings. First, it is unclear whether citizens’ trust in each medium is independent of the trust they have toward the others. In a modern, complex media landscape, it is hard to determine how the attitudes toward a particular medium can be disentangled from attitudes toward others. Second, there is no evidence that people actually possess specific opinions about each medium, and that they are equally thought through. It would be more reasonable to expect that their trust levels would be generally directed at the entire media they use as their source of information. Lastly, it is also unclear how comparable these measures are. For instance, will a 10-point drop in trust in one medium be equivalent to a 10-point drop in another? Will this similarity hold across context and time? And more fundamentally, is there a baseline level of trust for each of these media?

This division helps in more than merely identifying the unit of analysis of the trust object. It also reflects the envisioned societal role of the news media. If it is understood as being limited to delivering professional and accurate information to large audiences, then a micro- or mezzo-level perspective is in order. In contrast, if it is seen as a social institution, a wider perspective is required. The news media is the *fourth estate*, part of the democratic infrastructure, which includes institutions and processes that enable citizens to effectively participate in democracy (Coleman 2012; Exoo 2009). More evidence in support of this view is in the strong and persistent correlation between trust in the news media and trust in political institutions (Carr et al. 2014). Yet if trust in the news media only expresses approval of the integrity of sources, stories or the media that broadcasts them, it should be independent of approval of political institutions’ performance.

The solution proposed in this paper is to shift to the macro-level perspective and understand the news media as a social institution. Similar to other institutions, citizens use different aspects of the news media, sometimes many of them combined, and their attitudes toward the news media is based on how much trust they have in any combination of stories, sources and media they are exposed to, whether legacy or new. Measurement invariance of three hypothesized models will be tested. The first will group all media sources together and test whether they all load consistently into a single factor. The remaining two will test a hypothesized bifactorial structure, where media sources are partitioned into legacy versus online sources and news-producing versus web 2.0 apps used to disseminate news produced by other sources. Measurement invariance of any of these hypothesized models will be evidence for its cross-context comparability, and provide a valid proxy for the general public trust in news media.

## Measurement invariance (MI)

The ability to compare across context is a bedrock principle in cross-cultural research, which typically engages with comparing the attitudes, opinions and behaviors of heterogenous groups of individuals, grouped along spatial, time, class or cohort lines. Establishing comparability is necessary to avoid systematic bias due to group membership (Bollen 1989). The central aim in testing for MI is to determine whether a measurement model possesses the same psychometric properties across heterogeneous contexts (Vandenberg and Lance 2000). In other words, whether the same factorial structure underlies a set of manifest variables and latent construct(s), and whether equivalent associations between different latent constructs and observed variables are equivalent across different times and cases (Davidov et al. 2014; Wu and Estabrook 2016).

To test the measurement equivalence of trust in the news media, I employed multiple-group confirmatory factor analysis (Bollen 1989; Jöreskog 1971). MGCFA is a useful tool in the family of structural equation modelling, employed to assess the validity of measurements of latent constructs across contexts (Bollen 1989; Vandenberg and Lance 2000). Construct validity will be established by exploring the variation and co-variation of trust in different media, and identifying the number and nature of latent variable(s) that account for this variation. Unlike other methods (e.g., exploratory factor analysis) which are explorative in nature and aim at identifying inter-correlation between indicators as well as the minimal number of unique factors to explain these correlations, MGCFA is a theory-guided process, designed to allow testing hypotheses and meaningfully compare measures. Using MGCFA will allow assessing the equivalence of any measure of trust in the news media and subsequently determine its cross-case comparability.

Measurement invariance is established in three steps. First, the configural invariance of the model must be validated. This type of equivalence is sometimes referred to as ‘pattern’ or ‘baseline’ invariance (Steinmetz et al. 2009). Configural invariance means that the overall factor structure stipulated by the measure, fits well for all sample groups. In simple terms, a measurement would be configurally invariant if the same survey items would be useful in measuring the latent construct and load on the same factors across all administrations (Vandenberg and Lance 2000). A configurally invariant measure of trust in the news media would mean that the basic meaning and structure of trust in different news media—legacy as well as online—exists in all sample countries and loaded into an identical factorial structure. If measures are found non-invariant configurally, it may reflect cross-context differences in the numbers of factors item loading scores across cases, which would indicate that it cannot be used comparatively. In contrast, under configural invariance, we should expect that citizens’ judgements of the various stories, sources and media to be strongly related to each other, and that together they would adequately capture citizens’ latent trust in the news media.

While factors may be structured similarly, the magnitude of their loadings, i.e., their individual contribution to the latent construct, may vary with context, which would make it impossible to compare the results across cases. Therefore, the next step will be to test the model’s metric invariance (or ‘weak invariance’), which builds upon configural invariance by requiring, in addition to the construct being measured by the same items, that the factor loadings of those items will be identical across cases (Brown 2015). Failing to establish metric invariance may indicate that although the same media sources are relevant in people’s overall trust calculus across cases, the relations between the different factors are conditioned by context and therefore are incomparable across cases. In contrast, metric invariance would indicate that a one-unit change in trust in one country will be comparable to a one-unit change in all others. Establishing this type of invariance would suggest that the construct has the same meaning to participants across groups, and it would indicate that cross-cultural comparisons of covariances and unstandardized regression coefficients are possible (Bollen 1989; Byrne et al. 1989).

The last step is testing for scalar invariance (or ‘strong invariance’), which requires that item intercepts will also be equivalent across administrations. Scalar invariance allows mean comparisons across groups and when obtained, cross-group differences in the means of the observed items are interpreted as a result of differences in the means of their corresponding latent constructs (Meredith 1993). Scalar invariance is a quality that can be approximated at best (Marsh et al. 2018), as it is unattainable in many cases. In such cases, analysts are satisfied with partial scalar invariance, if at least two indicators have invariant intercepts across all groups (Brown 2015; Byrne et al. 1989). Ascertaining scalar invariance allows substantiating multi-group comparisons of factor means (e.g., *T*-tests or ANOVA), and provides confidence that any statistically significant differences in group means are not due to differences in scale properties in different groups (Bollen 1989; Jöreskog 1971; Vandenberg and Lance 2000).

## Case selection and data

Establishing the validity of any generalized measure of trust in the news media requires testing its equivalence for citizens living in societies with sufficiently different media landscapes, that are nonetheless culturally and politically close enough to make such comparisons valid. Moreover, any cross-case comparison of trust in the news media will only be meaningful if it pertains to cases with similar media landscapes, consumed under similar political, social and economic circumstances. The European Union (EU) is an ideal collection of cases to test the feasibility of measuring trust in the news media invariantly, thanks to the different historical, social and political circumstances of its Member States on the one hand, and their shared political institutions and commitment to a set of agreed upon values and principles on the other.

The data used in the analyses were collected in the framework of the Flash Eurobarometer study 464 (European Commission 2018). This timeframe was chosen to make sure that respondents’ attitudes were not affected by the unusual Covid-19 circumstances. The survey included data collected in all 28 EU Member States (including the UK, as data were collected prior to the completion of Brexit). Data about the attitudes and preferences of at least 1000 citizens were collected in all EU member states except Cyprus, Luxembourg and Malta, where the sample was smaller and included roughly 500 respondents per member state. In total, some 26,576 respondents making a representative sample of the EU population aged 15 or over were interviewed. This study included a battery of questions about trust in different media sources, asking ‘How much do you trust or not the news and information you access through: (1) Printed newspapers and news magazines, (2) Online newspapers and news magazines, (3) Online social networks and messaging apps, (4) Television, (5) Radio and (6) Video hosting websites and podcasts’. Four-point Likert scales, ranging from ‘do not trust at all’ to ‘totally trust’ were used to gauge the level of trust in each medium, with an additional ‘DK/NA’ category for respondents unable or unwilling to answer.

Table 1 shows the summary statistics for the study variables. One clear finding is that different media enjoyed varying levels of trust. Legacy media, with radio first and foremost, followed by television and newspapers were most trusted by respondents. The median score was 3 for news-producing media and 2 for video hosts and social networks. Lastly, item response rates were rather low. While tolerable with legacy media, for online platforms nonresponse rates were 27–34%. Listwise deletion would lead to losing over half of the observations, and imputations were impossible due to the ordinal-level of the original survey items. Since data are missing at random, I employed pairwise deletion in the analyses, which resulted in analyzing information from 26,380 respondents.

**Table 1:** Trust in different news media

Variable	*n*	Valid %	Missing %	Mean	SD	Median	Min	Max
Country	28	28	–	–	–	–	–	–
Radio	26,576	88.1	11.9	2.93	0.71	3	1	4
Television	26,576	94.9	5.1	2.78	0.75	3	1	4
Printed newspapers	26,576	86.9	13.1	2.75	0.75	3	1	4
Online newspapers	26,576	73.5	26.5	2.58	0.76	3	1	4
Video/podcasts hosts	26,576	65.6	34.4	2.23	0.78	2	1	4
Social networks and apps	26,576	72.5	27.5	2.22	0.78	2	1	4

*Data source* Flash Eurobarometer 464: Fake news and disinformation online, February 2018. Accessed via GESIS Data Archive: ZA6934, dataset version 1.0.0 (2018)

## Competing measurement models for trust in news media

**Fig. 1:**
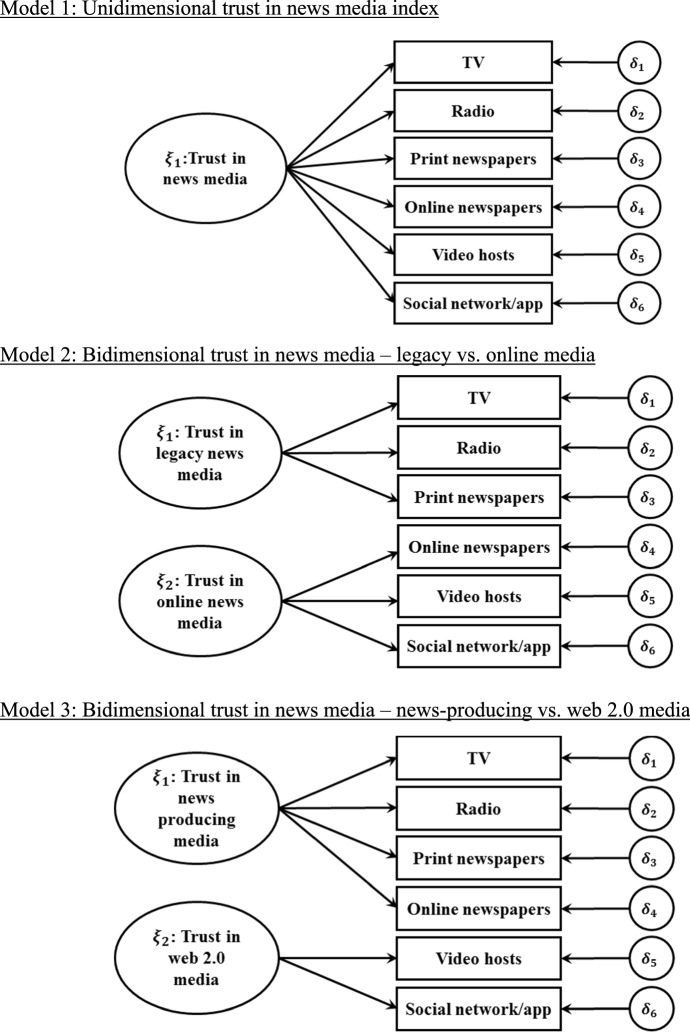
Hypothesized measurement models for trust in news media

Path diagrams with three hypothesized trust in news media measurement models are presented in Fig. 1. In the first model, trust in all six media types is hypothesized to be part of a single latent construct, namely news media trust. The rationale behind this model is that every citizen is exposed to a unique combination of stories, sources and media. Some of these, such as radio news, are considered by many as highly reliable, while others, such as social media apps, are seen by many as noncredible (Kiousis 2001; Newman et al. 2018). The attitudes citizens have toward their news media is based on this rich input as well as on their personal dispositions and experience. This combined input is at the basis of their trust calculus, which determines whether or not the media is generally carrying out its social role adequately.

The next two models propose that media trust is driven by two constructs. In model 2, the split is between legacy media and online media. The rationale is that while the public is exposed to various media, there is a qualitative difference in the news reports offered by each of them (Kiousis 2001; Ladd 2012). This distinction is evident considering the consistent differences in levels of trust expressed toward each. While legacy media enjoy higher levels of trust, online news is met with much skepticism (Fletcher and Park 2017; Kalogeropoulos et al. 2019; Tsfati and Cappella 2003). Model 3 slightly modifies model 2, by distinguishing online newspapers from other online, digital-born media, and grouping it together with legacy media in a group that creates news, as opposed to the digital-born media, who typically distribute news created by other media or generated by users. This is justified in light of the differences in median trust scores reported in Table 1, which shows that digital-born media enjoy lower levels of trust compared to all others.

These measurement models were fitted using a multigroup confirmatory factor analysis, where the vector of the observed trust variables $y$ was assumed to be caused by a vector of latent variables $\xi$, where $y$ contains ${y}_{i} (i=1,...,n)$ random variables and $\xi$ contains ${\xi }_{j} (j=1,...,m)$ common factors. The causal relationship between an observed variable ${y}_{i}$ and latent construct ${\xi }_{j}$ is represented in the regression equation:

1 $${y}_{i}={{\tau }_{i}}+{{\lambda }_{i}{\xi }_{j}}+{{\delta }_{i}},$$

where ${y}_{i}$ is the observed measure of trust, ${\xi }_{j}$ is the underlying latent factor causing the observed trust item ${y}_{i}$, ${\lambda }_{i}$ is a factor loading linking ${y}_{i}$ and ${\xi }_{j}$, ${\delta }_{i}$ is the residual variance in ${y}_{i}$ that is unexplained by ${\xi }_{j}$, and ${\tau }_{i}$ is the item intercept for ${y}_{i}$ (i.e., its value when ${\xi }_{j}=0$). This model implies that ${y}_{i}$ is a manifest indicator of ${\xi }_{j}$. Assuming that $E\left({y}_{j}\right)={k}_{j}$, $E\left({\xi }_{j},{\delta }_{i}\right)=0$, and that $E\left({\delta }_{i}\right)=0$, the expected value of ${y}_{i}$ can be expressed as:

2 $$E\left({y}_{i}\right)={\mu }_{i}={\tau }_{i}+{\lambda }_{i}{k}_{j}$$

The model implied covariance matrix of the $y$ observed variable caused by the latent variable $\xi$ is:

3 $$\Sigma =\Lambda \Phi \Lambda +{\Theta }_{\delta },$$

where $\Sigma$ is a $p\times p$ variance–covariance matrix among the $y$ observed variables, $\Lambda$ is a $p\times m$ matrix with items’ factor loading ${\lambda }_{i}$ on factor ${\xi }_{j},$ where $m$ is the number of common factors, $\Phi$ is an $m\times m$ variance–covariance matrix among the common factors in $\xi$, and $\Theta$ is a $p\times m$ diagonal matrix containing the residual variance ${\delta }_{i}$.

### Trust in news media as a unidimensional index

Invariance in each model was tested using the Maximum Likelihood estimator. For each baseline model, modification indices and expected parameter changes (EPC) were used and constraints were relaxed by introducing covariances between trust items, unrelated to the latent construct. Factor loadings greater than 0.3 were considered satisfying and the goodness-of-fit of each model was calculated using global fit statistics (Brown 2015). These included the Chi square statistic, the comparative fit index (CFI) and the Tucker–Lewis index (TLI). Additionally, two absolute-fit indices were obtained, including the root mean square error of approximation (RMSEA) and the standardized root mean square residual (SRMR). For an acceptable model fit, CFI and TLI must be greater than 0.95 (Bentler 1990; Bollen 1989), RMSE must be below 0.08 (Hu and Bentler 1998, 1999) and SRMR must be below 0.05 (Jöreskog and Sörbom 1996). Lastly, models will be considered unsatisfactory if their CFI drops by over 0.01 between steps, RMSE grows by more than 0.015 or SRMS grows by more than 0.01 (Chen 2007).

In model 1, trust in all six media, legacy and online, was loaded on a single, unidimensional factor. As shown in Table 2, the baseline model was found unsatisfactory. While CFI and SRMR were acceptable, the other two indices did not reach the acceptable thresholds. Following the modification indices analysis, five covariance terms were added to the model, accounting for dependencies between radio and television, radio and print newspapers, television and print newspapers, television and online newspapers and video/podcast hosts and social network apps. The modified model was satisfactory, with CFI and TLI reaching almost 1 and absolute measures falling well below the cut-off points. Next, cross-case configural invariance was tested, to establish that the factor loading structures are identical across cases. As indicated by the fit measures, this model was satisfactory, with all fit indices surpassing the required threshold. In substantive terms, establishing the configural invariance of this model means that all six items were related to the latent factors and they loaded to the same single factor across all cases.

**Table 2:** Trust in news media as a unidimensional measure

Model	Chi-sq	df	CFI	ΔCFI	TLI	ΔTLI	RMSEA	ΔRMSEA	SRMR	ΔSRMS
Overall fit	597.756	5	0.972		0.916		0.095		0.028	
Overall fit modified*	145.320	4	0.997		0.988		0.037		0.010	
Configural invariance	401.951	112	0.993		0.974		0.052		0.015	
Metric invariance	864.827	247	0.985	0.008	0.975	− 0.001	0.052	0.001	0.035	− 0.020
Scalar invariance	4612.182	382	0.898	0.088	0.887	0.087	0.108	− 0.057	0.080	− 0.046
Partial scalar invariance**	1186.217	274	0.978	0.007	0.966	0.008	0.059	− 0.008	0.038	− 0.003

The results presented in the Table were obtained by means of confirmatory factor analysis. Cell entries correspond with incremental and absolute fit indices in each of the three models. Changes in fit indices between invariance models are presented for CFI, TLI, RMSEA and SRMS

*Model modifications included: Radio ↔ television, print newspapers; Television ↔ print, online newspapers; Video hosts ↔ social network apps,

**Model modifications included: Radio, television, print newspapers, online newspapers ≁ 1

where:

↔ Indicates covariance residual between two observed variables;

≁Indicates relaxing equality constraints on the listed intercepts

*Data source* Flash Eurobarometer 464: Fake news and disinformation online, February 2018

Moving beyond the configural structure of the data, metric invariance was estimated next. Forcing equivalence on the factor loadings across cases was found justifiable, with acceptable fit measures and mostly acceptable changes in them, compared to the configural model. In one of them, namely SRMS, the estimate change was − 0.02, twice the acceptable threshold of − 0.01. However, given that the estimates for all fit indices were in the acceptable range, all other change metrics were also within the acceptable magnitude, and all standardized factor loadings exceeded 0.3, it is reasonable to interpret these findings as indicating that this model is metrically invariant. In substantive terms, this finding indicates that not only are all items related to the latent factor, but that the strength of these relations is consistent across all administrations.

Testing for scalar invariance returned unsatisfactory results. Both incremental fit indices fell below 0.9 and both absolute indices exceeded their acceptable cut-off points. This means that in its basic form, with all intercepts constrained, the model is too restrictive and does not allow cross-case comparisons of the means. This is because there is little certainty about whether differences between populations reflect different levels of trust or rather indicate that some populations responded to the measures differently than others. Relaxing some constraints by allowing some intercepts to vary helped establish partial-scalar invariance. When the intercepts for trust in television, radio, and both print and online newspapers were released, all four goodness-of-fit measures were well over their thresholds. Compared to the metric invariance step, changes in fit measures were moderate and all with acceptable magnitudes. Examining the country residual correlation matrices corroborated the good fit, with no residual larger than 0.1.

The model solution, corresponding with the hypothesized model in Eq. 1, is presented in the matrix below, where the latent structure ${\xi }_{1}$ is the unidimensional news media trust, ${\tau }_{1-4}$ are the non-constrained intercepts, ${\delta }_{i}$ are the error terms and the resulting y’s labels correspond with television, radio, print newspapers, online newspapers, video and podcast hosts and social network applications.

4 $$\left[\begin{array}{c}{y}_{TV}\\ {y}_{RD}\\ {y}_{NP}\\ {y}_{ON}\\ {y}_{VD}\\ {y}_{SN}\end{array}\right]=\left[\begin{array}{c}{\tau }_{1}\\ {\tau }_{2}\\ {\tau }_{3}\\ {\tau }_{4}\\ 2.585\\ 2.514\end{array}\right]+\left[\begin{array}{c}0.577\\ 0.525\\ 0.638\\ 0.857\\ 0.477\\ 0.464\end{array}\right]{\xi }_{1}+\left[\begin{array}{c}{\delta }_{1}\\ {\delta }_{2}\\ {\delta }_{3}\\ {\delta }_{4}\\ {\delta }_{5}\\ {\delta }_{6}\end{array}\right]$$

These results mean that the unidimensional measure of trust in the news media is comparable across contexts. Researchers using this measure can assume that it validly gauges people’s trust in the news media, and that cross-group mean differences reflect actual differences in latent factor means. A standardized, unidimensional index was created to validly reflect EU citizens’ trust in their news media. This index is fully comparable across all 28 member states and provides a unique opportunity for researchers interested in incorporating trust in the news media in their cross-cultural analyses, either as a dependent variable for researchers motivated by explaining what makes some groups in the population more trusting compared to others, or as an explanatory factor, to be used in models predicting another social or political outcome.

**Fig. 2:**
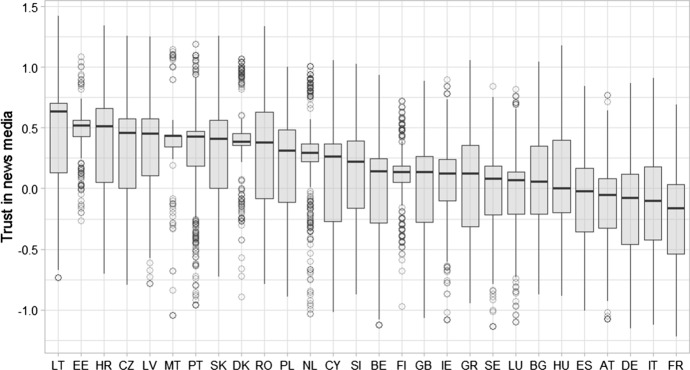
Cross-country distributions for trust in news media, unidimensional, partially invariant index. This figure shows per-country boxplots for the trust in news media index. The index was calculated based on the partially invariant unidimensional model, for all survey respondents in all countries that provided valid responses on their trust in all types of media (*N* = 13,065)

Figure 2 shows the cross-case locality, spread and skewness of the index, which ranged between − 1.22 and 1.44. The grand mean was 0.1 (with a standard deviation of 0.43 and a grand median of 0.14). The probability distribution was slightly skewed to the left (− 0.43), indicating that EU citizens’ trust in the news media is positive overall, as also reflected in the overall median per-country, which was positive in 23 out of the 28 cases. The kurtosis was 0.05, suggesting that the deviations from the mean were not extreme. While these results are based on cross-culturally equivalent measures, significant variations across countries is observed. This indicates that context is likely to play an important role in people’s trust in the news media, and modeling trust must account for cross-case differences. Testing for intra-cluster correlation indicated that roughly 30% of the variance in this index is attributed to the context respondents experience, likely their cultural, political and economic environment, as well as news media supply side differences across cases.

Three further findings are presented in Fig. 2. First, the news media enjoyed the trust of 75% or more of the public in Croatia, the Czech Republic, Denmark, Estonia, Finland, Latvia, Lithuania, Malta, the Netherlands, Portugal and Slovakia. In the remaining 17 countries, 25% of the population or more expressed negative attitudes, while 25% or more indicated that they trust the media. In contrast, the majority in the Austrian, French, German, Italian and Spanish populations did not trust the news media, and in 13 other countries, namely Hungary, Bulgaria, Luxembourg, Sweden, Greece, Ireland, the UK, Finland, Belgium, Slovenia, Cyprus, Poland and Romania, 25–50% of the population had negative attitudes to the media. Lastly, relative homogeneity of attitudes toward the media was observed in Estonia, Malta, Denmark, the Netherlands and Finland, while in most other countries there was much more variance. Outliers were observed in countries with relatively homogenous distributions.

### Trust in news media as a bidimensional index

The next two models tested whether a measure of trust in the news media would better fit a bifactorial structure. In model 2, trust is partitioned between two hypothesized factors: legacy and new/online media. The results from this model are presented in Table 3. We can note that all but one fit measure in the initial overall fit model were unacceptable and required modifications. Specifically, covariation terms were added between radio and television, print and online newspapers, and video hosts and social media apps. This model proved configural and metric equivalence, with an unsatisfying change in SRMS between the two steps, similar to what was observed in model 1. None of the fit measures yielded satisfying results testing scalar invariance and multiple efforts to obtain partial-scalar invariance have failed. The best result was obtained after relaxing the intercept restrictions on television and online newspapers, but the change in incremental fit measures compared to the metric invariance model was too large to accept the results as indicating equivalence. In sum, the bidimensional partition into legacy and online media yielded a metric invariance but not a (partial-) scalar one.

**Table 3:** Trust in news media as a bidimensional measure—legacy vs. online media

Model	Chi-sq	df	CFI	ΔCFI	TLI	ΔTLI	RMSEA	ΔRMSEA	SRMR	ΔSRMS
Overall fit	2831.221	8	0.934		0.877		0.116		0.044	
Overall fit modified*	69.214	5	0.999		0.996		0.022		0.006	
Configural invariance	432.577	140	0.993		0.979		0.047		0.014	
Metric invariance	733.014	248	0.988	0.005	0.980	− 0.001	0.046	0.002	0.029	− 0.015
Scalar invariance	3244.461	356	0.930	0.058	0.917	0.063	0.093	− 0.047	0.059	− 0.030
Partial scalar invariance**	1302.019	302	0.976	0.012	0.966	0.014	0.059	− 0.014	0.036	− 0.007

The results presented in the Table were obtained by means of confirmatory factor analysis. Cell entries correspond with incremental and absolute fit indices in each of the three models. Changes in fit indices between invariance models are presented for CFI, TLI, RMSEA and SRMS

*Model modifications included: Radio ↔ television; Print newspapers ↔ online newspapers; Video hosts ↔ social network apps,

**Model modifications included: Television, online newspapers ≁ 1

Where:

↔ Indicates covariance residual between two observed variables;

≁Indicates releasing of equality constraints on the listed intercepts

*Data source* Flash Eurobarometer 464: Fake news and disinformation online, February 2018

Lastly, the bidimensional model partitioning trust in news media between news-producing and web 2.0 media obtained similar results. As shown in Table 4, the initial overall model fit measures were unsatisfying, and covariance terms between trust in radio and television, and video/podcasts hosts and social network apps were introduced. All fit indices were satisfactory in the modified overall model and remained so in the next one, indicating configural invariance. The changes in three fit indices between the configural and metric model were acceptable, while the change in RMSEA was slightly higher than acceptable (0.02). However, given the strong fit identified by the other indices, we can reasonably conclude that this partition into two factors yields metric invariance. The scalar model returned mixed results, with only the absolute fit indices reaching the threshold. Changes in these indices were beyond the acceptable cutoff point, which made the scalar model invariant. Relaxing some of the intercept constraints did not help in establishing partial scalar invariance, as although all fit measures reached the required threshold, their values changed substantially from the metric model. Thus, we can conclude that similar to the previous bidimensional model, while metric invariance was found, scalar invariance was not.

**Table 4:** Trust in news media as a bidimensional measure—news-producing vs. web 2.0 media

Model	Chi-sq	df	CFI	ΔCFI	TLI	ΔTLI	RMSEA	ΔRMSEA	SRMR	ΔSRMS
Overall fit	2808.535	8	0.935		0.878		0.115		0.044	
Overall fit modified*	168.612	5	0.996		0.989		0.035		0.011	
Configural invariance	540.382	140	0.990		0.971		0.055		0.017	
Metric invariance	899.927	248	0.984	0.006	0.973	− 0.002	0.053	0.002	0.031	− 0.014
Scalar invariance	2502.684	356	0.948	0.036	0.939	0.035	0.080	− 0.027	0.048	− 0.017
Partial scalar invariance**	1652.566	302	0.967	0.017	0.954	0.019	0.069	− 0.016	0.040	− 0.009

The results presented in the Table were obtained by means of confirmatory factor analysis. Cell entries correspond with incremental and absolute fit indices in each of the three models. Changes in fit indices between invariance models are presented for CFI, TLI, RMSEA and SRMS

*Model modifications included: Radio ↔ television; Online newspapers ↔ Video hosts, social network apps,

**Model modifications included: Radio, television ≁ 1

Where:

↔ Indicates covariance residual between two observed variables;

≁Indicates releasing of equality constraints on the listed intercepts

*Data source* Flash Eurobarometer 464: Fake news and disinformation online, February 2018

## Discussion and conclusions

This study set out to answer to what extent can the measurement invariance of trust in the news media be established for individuals living in 28, as of 2018, EU Member States. It makes a contribution to the literature on media trust, and specifically to the debates around the conceptualization and measurement of this construct. While this literature often employs various measures of trust in comparative analyses, these measures are often not validated and the scarce attempts to validate them have led to unsatisfying results, providing little ground for comparative analyses. Conceptual and measurement debates still exist in the literature, despite century-long efforts to resolve them. The issue becomes even more important with the changes in the media landscape and the rise of public sentiments critical of social institutions in general and the news media in particular. This sense of crisis is not limited to trust in the media and extends to trust in multiple social and political institutions (Bogaerts and Carpentier 2013; Coleman 2012).

The analysis employed salient conceptualization and used multi-group confirmatory factor analysis to test whether or not three competing measurement models are comparable across different EU Member States. The tests aimed at establishing equivalence in these models’ factorial structure, loadings and intercepts. The findings showed the pertinence in conceptualizing and measuring news media trust using a unidimensional approach, where trust in various media is assumed to be caused by the same latent construct. These findings suggest that it would be more appropriate to understand trust in the news media as an assessment people make of the entire corpus of the news media they are exposed to. While some news media sources enjoy higher levels of public trust, no evidence was found to suggest that people establish their trust in news sources they consume while differentiating between them based on their reputation.

The findings are also encouraging with respect to the potential of large cross-national research on the correlates of trust in news media. Establishing the unidimensional partial scalar invariance of the measurement, suggests that the factors loading structure, the loading values and the values of two of the intercepts do not systematically depend on irrelevant aspects of data collection such as sampling, translation issues, different interpretation of questions, or cultural difference in social desirability and acquiescence. Rather, the levels of trust in news media observed using this index are casually related to the latent scores and can be incorporated into a factor with identical structure, loadings and partially identical intercepts to accurately reflect the real level of trust EU citizens have in their news media.

These findings imply that despite the clear qualitative differences between different media, the level of trust the public has in the news media is based on a calculus that accounts for all media sources combined. When survey respondents are asked about their trust in the media, their answers are not based on the net, individual qualities of each medium, reporter or story. Rather, their trust is informed by the general trustworthiness of the news media they are exposed to. While many respondents may be able to consider media micro-level performance, comparing their levels of trust across cases requires factoring their trust in different media using a unidimensional index. Additionally, while trust in web 2.0 media proved to be constrainable across cases, the cross-case variance between different legacy media was more prominent and cannot be restricted. While this result does not condition the relationship between the latent construct and these four items, it will affect the cross-case variation in mean levels of trust.

The results also indicate that the bidimensional measurement models were metrically invariant, but their scalar invariance, or part of it, was not attainable. The invariance found in these models indicates that people in different EU countries responded to the items in the same way, so that the strength of relations between specific scale items and their respective latent constructs are the same across context. This means that the ranking of different media according to the trust levels they enjoy in the population, as often done by the Eurobarometer, is comparable across countries, and observed item differences will indicate country-differences in the underlying construct. Grouping these items into two dimensions, however, does not constitute a valid measurement model and will lead to biased estimates. Bifactorial measures of trust in the news media must not be employed when testing theories about the sources or consequences of media trust and its cross-context variance.

In this rapidly changing media scene, it is easy to understand why many see the differences between legacy media and online media as two different things. The professionalism, accuracy and fair attitude of legacy media are incomparable to the eclectic and sometimes chaotic nature of online news, and with these differences one may expect that people’s judgement of the media will be differential. A similar argument can be made about the qualitative differences between tabloids and spreadsheets, or between news on a TV morning show and the evening news. However, the modern media environment presents users with hard news and kitten images, all blended into one feed. Many users are aware of these differences, yet they assess the news media as a whole, a summary of all news they are exposed to, pertaining to any story, delivered by any source and disseminated by any medium. This way of judging the media is common to citizens across the EU, despite the grave differences in how the media performs under specific political, economic and social contexts.

This study has two main limitations which provide opportunities for future research. First, the analyses are based on cross-sectional data, which does not allow establishing the invariance of the measures across time, and therefore more research is needed to validate the longitudinal unidimensionality of media trust. Second, these data come from a single area in the world, the EU. While focusing on a collection of countries with cultural and political similarities makes sense for establishing measurement invariance, future research would benefit from extending the selection of cases and testing the model equivalence in additional cases to establish its universality..

The alarming warnings about the decline in media trust and the strong partisan divide that drives them are serious. The term ‘fake news’ is no longer limited to participants in political rallies. It has become part of modern society. The warnings are serious because the trust people have in the media is much more than their mere approval of the quality of reporting. Similar to trust in any other social institution, trust in the news media involves risk. The risk is not limited to the trustor, who may be misinformed and consequently participate in democracy less efficiently. It affects the trustee, the news media itself, which is seen by many as not carrying out its main role of supporting democratic society. While having a critical attitude to social institutions is good for democracy as it allows citizens to communicate their critique and allows institutions to improve, the danger at higher levels and with rapid deterioration is that media critique may transform into media cynicism, with unwanted consequences for modern society and liberal democracy.

## Supplementary Information

Below is the link to the electronic supplementary material.

Supplementary file1 (R 3 kb)

## Data Availability

Flash Eurobarometer 464: Fake News and Disinformation Online (v1.00). (2018). [Data set]. European Commission, Directorate-General for Communication. http://data.europa.eu/88u/dataset/S2183_464_ENG

## Code availability

The code used for the analyses is available in the supplementary material.
